# Application of Pulsed Electric Fields for Obtaining Antioxidant Extracts from Fish Residues

**DOI:** 10.3390/antiox9020090

**Published:** 2020-01-21

**Authors:** Daniel Franco, Paulo E. S. Munekata, Rubén Agregán, Roberto Bermúdez, María López-Pedrouso, Mirian Pateiro, José M. Lorenzo

**Affiliations:** 1 Centro Tecnolóxico da Carne de Galicia, rúa Galicia n° 4, Parque Tecnolóxico de Galicia, San Cibrao das Viñas, 32900 Ourense, Spain; danielfranco@ceteca.net (D.F.); paulosichetti@ceteca.net (P.E.S.M.); rubenagregan@ceteca.net (R.A.); robertobermudez@ceteca.net (R.B.); mirianpateiro@ceteca.net (M.P.); 2 Department of Zoology, Genetics and Physical Anthropology, University of Santiago de Compostela, 15872 Santiago de Compostela, Spain; mariadolores.lopez@usc.es

**Keywords:** sea bream, sea bass, by-products, antioxidant capacity, amino acid

## Abstract

Fish processing has serious economic and environmental costs in the food supply chain. It is necessary to find new ways to convert fish residue to added-value products, especially for main aquaculture species. In this study, a pulsed electric field (PEF) process for antioxidant extract production from three residues (gills, bones, and heads) of two commercial species (sea bream and sea bass) was tested. Three methods of extraction using two solvents (water and methanol) and a water extraction assisted by PEF were assessed. Chemical and mineral compositions, as well as amino acid profile of the by-products, were determined. In addition, four in vitro antioxidant methods, 2,2-diphenyl-1-picrylhydrazyl radical scavenging activity (DPPH), 2,2-azinobis-(3-ethyl-benzothiazoline-6-sulphonate radical (ABTS), ferric reducing antioxidant power assay (FRAP), and oxygen radical absorbance capacity assay (ORAC), were used to evaluate the extracts. Antioxidant activity was confirmed by DPPH and ABTS and FRAP tests, obtaining the highest values for residues from the sea bream species. ORAC values were higher in methanol than in water solvent. In general, gills were the residues with the greatest antioxidant activity for the four antioxidant assays employed. For DPPH assay, the extracts of water assisted by PEF from heads, bones, and gills yielded significant increases of 35.8%, 68.6%, and 33.8% for sea bream and 60.7%, 71.8%, and 22.1% for sea bass, respectively, with respect to water extracts. Our results suggest that PEF would be an environmentally friendly and economic choice for antioxidant-extract production from low-value by-products from fish processing.

## 1. Introduction

Aquaculture production has been globally increasing and gaining economic importance over the last decade. Particularly for the European market, the main cultured aquaculture species are the Atlantic salmon, rainbow trout, gilthead sea bream, European sea bass, common carp, and turbot [1]. Due to the large production of ready-to-use products and meals, the fish industry generates a large amount of wastes. These residues in fisheries are mainly composed of heads, skin, and viscera and account for 20–75% of fish weight [2]. In this sense, great efforts are being made to exploit these fish wastes, ensuring the sustainability of the aquaculture industry. This strategy is supported by the presence of natural antioxidants and other bioactive components in these residues.

It has been demonstrated that protein hydrolysates (FPH) obtained from fish by-products have an excellent quality in terms of amino acids composition and antioxidant properties [3,4]. In order to extract amino acids and small peptides with more bio-accessibility and bioavailability for health purposes, the use of heating, chemical, and enzymatic treatment have been reported [5]. However, enzymatic hydrolysis has a great cost in comparison to chemical hydrolysis, which is of very limited use at the industrial level [6]. Similarly, the other conventional technologies (thermal and chemical) have disadvantages related to extended process time, high energy consumption, and the use of toxic solvents.

On the other hand, emerging technologies (ultrasound, high hydrostatic pressure, ohmic heating, and pulsed electric fields, for instance) contribute to the extraction of bioactive compounds from foodstuffs. Particularly for pulsed electric fields (PEF), this technology consists of treating samples with high-voltage electrical pulses for short periods, which disturbs the structure of cell membranes and facilitate the extraction of entrapped bioactive compounds. Moreover, it was demonstrated that PEF treatment can also cause protein hydrolysis [5]. Extractions using PEF technology have been employed in plant [7], animal [8], and fish [9] products, but the scientific information about their use in the valorization of fish by-products is scarce.

Due to characteristics of food, the occurrence and progression of oxidative reactions generates the reactive oxygen species (ROS) by means of enzymatic, chemical, and photochemical reactions. Consequently, the formation of undesirable volatile and carcinogenic compounds is favored, along with changes in the functionalities of proteins, lipids, and carbohydrates, leading to the deterioration of sensory properties and reduced shelf life [10,11]. To tackle this problem, one of the most employed strategies in food systems is the use of antioxidants. Antioxidants are compounds that protect the lipid from oxidation and avoid the formation of rancid flavor and aroma in food products and also extend their shelf life. The synthetic ones are the most common antioxidant additives in food products (butylated hydroxyanisole (BHA), butylated hydroxyltoluene (BHT), propyl gallate (PG), and tert-butyl hydroquinone (TBHQ)). However, some physical properties of BHT and BHA, such as their high volatility and instability at elevated temperature [12] and their potentially harmful impact on human health, pressure governmental authorities to creation restrict rules in order to limit their use. Therefore, replacing synthetic antioxidants is becoming a main goal in the food industry [13].

There are abundant research studies about the use of natural antioxidants from plants, both terrestrial [14] and of marine origin [15], and their active molecules (polyphenols, ascorbic acid, carotenoids, tocopherol, and photosynthetic pigments) in foods. Although fish wastes were also considered to be sources of powerful antioxidants, there is less literature.

Taking into account that the PEF approach is an attractive strategy to valorize the residues from fish industry (because of its reduced time process, improved extraction yield, and environmental aspects) and fish by-products can offer interesting bioactive molecules, such as antioxidants (which can be applied in food industry), the aim of the present preliminary study was to evaluate whether sea bream and sea bass by-products (head, bone, and gills) treated with pulsed electric field technology provide antioxidant extracts.

## 2. Materials and Methods 

### 2.1. Samples

The sea bream and sea bass used were purchased from a local supermarket. By-products, heads, bones, and gills were manually obtained from the fishes. Then, these residues were chopped, vacuum-packaged, and stored at −20 °C, until further analysis.

### 2.2. Extraction Procedures

#### 2.2.1. Extraction with Solvents (Water and Methanol) without PEF

The heads, bones, and gills from sea bream and sea bass were extracted in a conventional manner, using water and methanol. Briefly, five grams of each one of the residues was mixed with 5 mL of distilled water or methanol. The mixture was intensively crushed and vortexed with an IKA T25 digital ultra-turrax (IKA^®^-Werke GmbH & Co. KG, Staufen, Germany), until complete homogenization. Then, ultrasounds were applied for 15 min at room temperature, to increase extraction yield. The obtained extract was centrifuged at 2000× *g* for 10 min, at 4 °C, and the resultant supernatant was passed through 45 µm pore-size filters (Filtros Anoia S. A., Barcelona, Spain). Extracts were stored at −20 °C until further analysis.

#### 2.2.2. Extraction with Pulsed Electric Fields (PEF)

**Figure 1:**
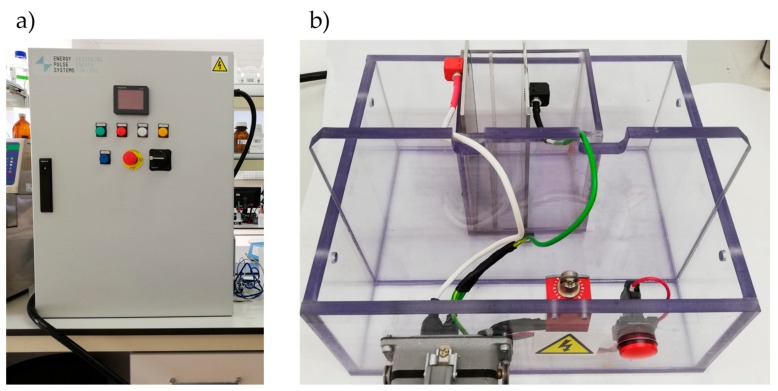
PEF generator (**a**) and the batch treatment chamber (**b**).

Fifty milligrams of each one of the residues from sea bream and sea bass, previously defrosted at room temperature, was weighed and mixed with 50 mL of distilled water. The mixture was intensively crushed and vortexed with an IKA T25 digital ultra-turrax (IKA^®^-Werke GmbH & Co. KG, Staufen, Germany) until complete homogenization. Then, the homogenates were placed between two electrodes separated by 5 cm, reaching 1.8 cm of height. PEF was generated by using a semiconductor-based positive Marx modulator Epulsus-PM1-10 equipped with a batch treatment chamber (EnergyPulse Systems, Lisbon; Portugal; Figure 1). The PEF working conditions were as follows: 7000 V potential difference, 20 µs pulse width, 10 Hz frequency, and pulses number of 100. Before starting the PEF treatment, the homogenates’ conductivity was measured in order to know the applicable voltage. The same electrical field was applied for all samples (1.40 kV/cm). The sea bream heads, bones, and gills achieved 26.9, 29.4, and 28.3 kJ/kg, respectively; meanwhile, sea bass head, bone, and gills achieved 26.6, 17.4, and 28.3 kJ/kg, respectively. The entire process was carried out protected from light. Once the PEF treatment was applied, the samples were extracted as described in Section 2.2.1. All treatments were made by triplicate.

### 2.3. Analytical Determinations

#### 2.3.1. Chemical Composition, Fatty Acid, Amino Acid, and Mineral Profile

The International Organization for Standardization (ISO) recommended standards were used to assess moisture [16], protein [17], and ash [18]. Total fat was extracted according to the American Oil Chemists Society (AOCS) Official Procedure Am 5-04 in an extractor Ankom XT10 (ANKOM Technology Corp., Macedon, NY, USA) [19]. Fatty acid extraction and identification was carried out with gas chromatography (GC-Agilent 7890B, Agilent Technologies, Santa Clara, CA, USA) with a flame ionization detector (FID) and PAL RTC-120 auto sampler, amino acid profile after protein hydrolysis employing high-performance liquid chromatography (Alliance 2695 model, Waters, Milford, MA, USA) with fluorescence detector (model 2475, Waters, Milford, MA, USA), and mineral composition was determined by induced coupling plasma atomic emission spectrometry [20].

#### 2.3.2. Determination of Antioxidant Capacity

##### DPPH Radical Scavenging Assay

The DPPH (2,2-diphenyl-1-picrylhydrazyl) scavenging method was carried out as follows [21]: the DPPH solution (60 μM in methanol) was mixed with 100 μL of sample. The mixture was incubated at 37 °C for 10 min and then the absorbance was measured in a spectrophotometer (UV-1800, Shimadzu Corporation, Kyoto, Japan) at 515 nm. Each extract was analyzed in triplicate, and its antioxidant activity was determined by using Trolox (Acros organics, Morris Plains, NJ, USA) as standard, expressing the results as μg Trolox/g sample.

##### ABTS Radical Cation Decolorization Assay

This method was determined according to the procedure previously described by Re et al. [22], with some modifications. The method is based on the decolorization of blue–green color at 734 nm, since ABTS radical ((2,2-azinobis-(3-ethyl-benzothiazoline-6-sulphonate) is scavenged. This radical was prepared by mixing 7 mM ABTS stock solution with 2.45 mM potassium persulfate and keeping the mixture in darkness, at room temperature, for 12–16 h before its use. The ABTS solution was added to 20 μL of sample, and the resultant mixture was mixed and left in darkness for 10 min. Afterward, absorbance was measured in a spectrophotometer (UV-1800, Shimadzu Corporation, Kyoto, Japan) at 734 nm. Each extract was analyzed in triplicate, and its antioxidant activity was determined by using a standard curve of ascorbic acid (AA) in the concentration range 0–150 mg/L AA, expressing the results as mg AA/100 g sample.

##### Ferric-Reducing Antioxidant Power (FRAP) Assay

The FRAP assay was based on the Benzie and Strain method [23], with some modifications. The FRAP reagent was prepared by using 0.3 M acetate buffer (pH 3.6), 10 mM 2,4,6-tripyridyl-s-triazine (TPTZ) in 40 mM HCl, and 20 mM FeCl_3_·6H_2_O solutions. These three solutions were mixed in a ratio of 10:1:1 (*v:v:v*). Afterward, 900 µL of this resultant FRAP solution was added to 30 μL of properly diluted sample and to 90 μL of distilled water. The mixture was heated at 37 °C and left to react for 20 min at this temperature. After this time, the absorbance was measured in a spectrophotometer (UV-1800, Shimadzu Corporation, Kyoto, Japan) at 593 nm. Each extract was analyzed in triplicate, and its antioxidant activity was determined by using a standard curve of FeSO_4_ in the concentration range 0–400 μM FeSO_4_, expressing the results as μmol Fe^+2^/100 g sample.

##### Oxygen Radical Absorbance Capacity (ORAC) Assay

The ORAC assay was determined according to the Huang et al. method [24], with some modifications. The reaction was carried out in 75 mM phosphate buffer (pH 7.4), for a final reaction volume of 200 µL. Then, 25 µL of sample was mixed with 150 µL fluorescein (80 nM of final concentration), and the mixture was pre-incubated at 37 °C for 30 min. Afterward, 25 µL of AAPH (2,20-azobis (2-methylpropionamidine) dihydrochloride solution (184 mM, final concentration) was added rapidly, using the injectors of a Synergy™ H4 Hybrid Multi-Mode Microplate Reader (BioTek Instruments, Inc., Winooski, VT, USA). The plates were immediately placed in the reader and the fluorescence was recorded every minute for 150 min and stirred prior to each reading (excitation wavelength, 485 nm, and emission wavelength, 528 nm). Eight calibration solutions, in the concentration range 0–100 μM, using Trolox as standard, were used in each assay. The phosphate buffer was used as blank. Each extract was analyzed in triplicate, and its antioxidant activity was calculated from the differences in areas under the fluorescein decay curve between the blank and the sample. The results were expressed as mg Trolox/g sample.

### 2.4. Statistical Analysis

Statistical analysis of the obtained data was conducted by using the IBM SPSS Statistics 23.0 program (IBM Corporation, Somers, NY, USA). An analysis of variance (ANOVA), using the General Lineal Model (GLM) procedure, was performed for chemical composition of residues. The following model was used:

Yij= μ+ Si + Dj + (S × D)ij + εij(1)

where, Yij is the observation of dependent variables, μ is the overall mean, Si is the effect of species, Dj is the effect of residues, (S × D)ij is the interaction term of species and residue, and εij is the residual random error associated with the observation. For extract antioxidant capacity, one-way analysis of variance (ANOVA) was applied to all assessed tests (DPPH, ABTS, FRAP, and ORAC), and the results were expressed as mean (±) standard error. The least square means (LSM) were separated by using Duncan’s post hoc test (significance level *p* < 0.05).

## 3. Results

### 3.1. Chemical, Mineral, and Amino Acids Composition of Fish Residues

In Table 1, the chemical and mineral composition from residues of sea bream and sea bass are depicted. Water content was the main component for the three by-products, with an average value of 55%, reaching a highest percentage above 60% in sea bass gills. However, there was a contradictory trend between species. In sea bass, gills showed the higher content than bones, with values of 62.37% and 51.60%, respectively. Conversely, bones showed higher water content than gills in sea bream (Table 1). In general, there were significant differences (*p* < 0.05) among by-products in both species, but water content in sea bream by-products was narrower (52.52–57.63%) than in sea bass.

Fat content showed values significantly higher (*p* < 0.05) in sea bass bone than gills and head; meanwhile, this trend was opposite in the sea bream. This fat composition is expected because there is a strong correlation between water and fat content. Within the fat content, MUFAs were the most abundant fatty acids, with values of approximately 40% of the total FAs, followed by PUFAs, with percentages around 30%. The gills presented the highest percentages of MUFAs, but the lowest of PUFAs, especially in the sea bass, and a significant (*p* < 0.05) increase of SFAs with respect to the other residues. Moreover, the gills showed the highest content in n3 fatty acid, as well as in long chain n3 fatty acids and therefore the most suitable n6/n3 (<1) ratio with respect to the others residues. The protein content presented antagonistic values according to the species, because bone reached the highest protein value (16.39%) in sea bream; meanwhile, bone reached the lowest value (14.24%) in the sea bass. Ash contents were more consistent between species; the ashes were more abundant in the head than in the rest of residues, reaching a value close to 10% in both species.

Regarding mineral profile, calcium highlighted over the rest samples with values even higher than 2000 mg/100 g residues in several parts (Table 1). An average value of 2448.2 mg/100 g was the highest content shown in the head from both species. Moreover, phosphorous is presented in substantial quantities, especially in the head, with values higher than 1200 mg/100 g residue in both species. Bones and gills were the most abundant source of potassium and sodium, with mean values of 281.7 mg/100 g and 254.6 mg/100 g residue, respectively.

The amino acid profile of the residues from sea bass and sea bream is depicted in Table 2. There were higher significant differences among residues than between species. For all residues, there was a lower content of essential amino acids compared to nonessential ones, showing EAA/NEAA ratios lower than 1 for all cases. In this sense, fish bones were more interesting and became healthier regarding this ratio, with average value of 0.87 (*p* > 0.05) for both species. On the contrary, gills provided the lowest ratio, with an average value of 0.67 (*p* > 0.05) for both species. Among essential amino acids, threonine, valine, lysine, and leucine were highlighted over the rest. The amount of threonine became more important in gills from both species. Specifically, the main essential amino acids were arginine, followed by leucine and lysine for the head and bones in both species. Wide variations were found in arginine, which presented significantly (*p* < 0.05) different values depending on the sample. The head and bone displayed values in the range 700–900 mg/100 g of residue, while the head was around 200 mg/100 g of residue. The sea bream bone showed the highest essential amino acid content. Concerning nonessential amino acids, the more predominant were glutamic acid, glycine, and aspartic acid in both species and residues.

### 3.2. Antioxidant Activity of EXTRACTs from Residues

**Figure 2:**
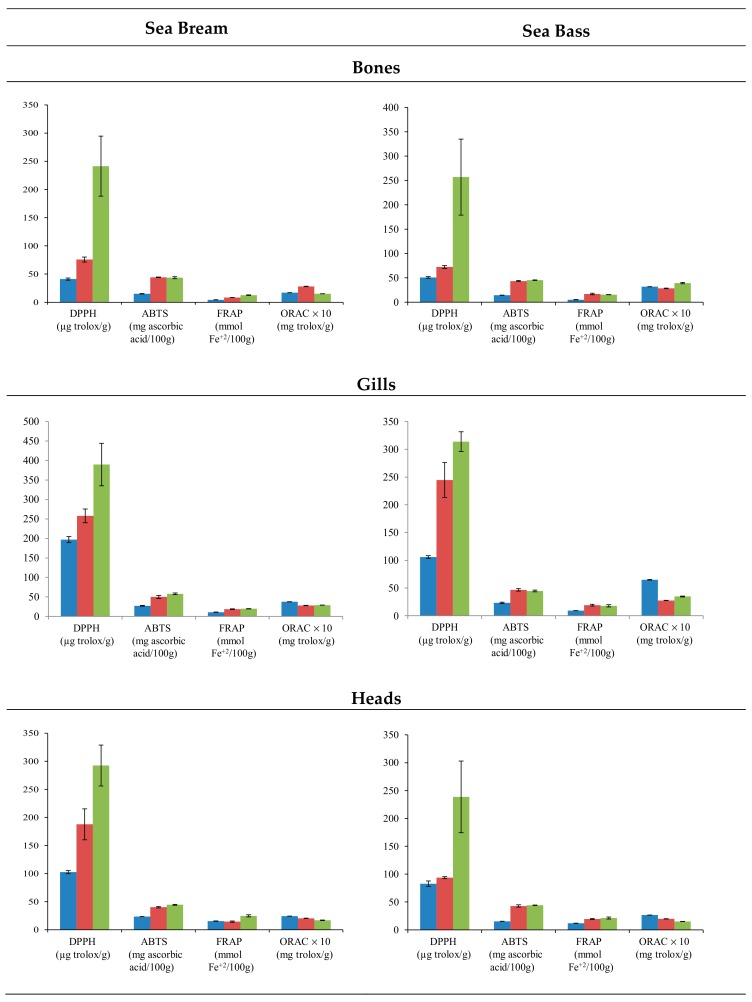
Mean values ± SEM. Antioxidant capacities in residues (bones, gills, and heads) of sea bream and sea bass, using methanol (blue), water (red), and water assisted by PEF (green) extractions.

Fish residues are a source of biological active peptides, with antioxidant-potential activity. To determine the antioxidant capacity of the extracts, the assays often included radical cation scavenging activity; specifically, the DPPH, ABTS, and FRAP assay evaluated the electron transfer capacity, and ORAC assay was employed to evaluate the proton transfer capacity. The use of different treatments affected the extraction of compounds with antioxidant activity in both species (Figure 2). In general, the application of PEF provoked the extraction of compounds with the higher antioxidant activity in all residues. Particularly, the DPPH test displayed significant differences (*p* < 0.001) in all residues analyzed except in sea bass gills. On the contrary, methanolic extraction provided the lowest results in terms of antioxidant activity, except for ORAC values. Regarding the different residues employed, the extracts of gills showed higher antioxidant activity. In the case of water assisted by PEF, values that exceeded 300 µg Trolox/g gills resulted in the DPPH test. On the contrary, bones extracts showed the lowest results with DPPH values that barely reached 250 µg Trolox/g bones in water extraction assisted by PEF. Specifically, the antioxidant capacity displayed higher values in gills (196.85–389.62 µg Trolox/g sample) and heads (102.75–292.47 µg Trolox/g sample) for sea bream than in gills (105.93–313.87 µg Trolox/g sample) and heads (82.79–238.76 µg Trolox/g sample) for sea bass. However, the antioxidant capacity of sea bass bones (50.78–256.78 µg Trolox/g sample) was higher than sea bream bones (41.18–241.43 µg Trolox/g sample). The differences among heads’, bones’, and gills’ antioxidant activities were more remarkable for the DPPH assay.

**Figure 3:**
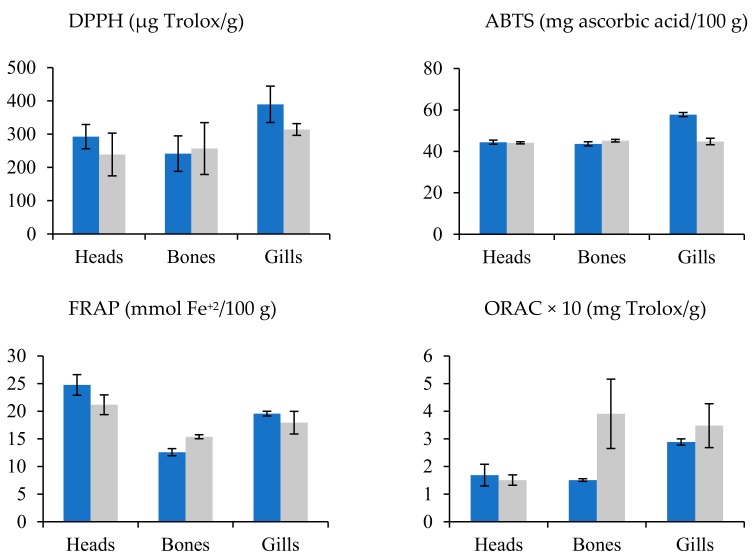
Mean values ± SEM. Antioxidant capacities in residues (bones, gills, and heads) of sea bream (blue) and sea bass (gray).

In the sea bream species, residue extracts presented higher antioxidant activity than sea bass residues. The solvent and residue variable showed remarkable differences, but the species did not show a clear trend for antioxidant capacity, above all (Figure 3). In this sense, water and methanol do not produce a clear trend in the activity of extracts. Conversely, water assisted by PEF clearly increased the antioxidant activity. Head and gill extracts from sea bream reached values higher than those from sea bass; meanwhile, the sea bass bones’ extract showed antioxidant activity values higher than those of the sea bream bones (Figure 2).

## 4. Discussion 

### 4.1. Chemical, Mineral, and Amino Acids Composition of Fish Residues

Fish-processing by-products are great sources of high-quality compounds, such as proteins, amino acids, fats, and others; consequently, the chemical composition is relevant information. As it can be expected, the water content was the principal component of other fish residues, as reported in the literature. Fat and protein content showed important differences in heads, gills, and bones between species. This high degree of chemical variability among species have been reported for other residues. In addition, intrinsic factors (age, sex, and life cycle) and extrinsic factors (temperature, salinity, and feeding) [25,26]. However, no data about chemical composition of gills were found in the literature. Regarding fatty acid composition, our results were expected, because fatty fishes, such as sea bream and sea bass, are rich in PUFA. This fatty acid group is represented by long-chain fatty acids (eicosapentanoic acid (EPA, C10:5n-3), docosahexaenoic acid (DHA, C22:6n-3)) and omega-3 fatty acids in the linolenic series [27]. Overall, differences in chemical composition among fish species are common, even from an individual fish to another [27].

It is well-known that fish products are highlighted for their great content of calcium and phosphorus, especially in bones [28], and this fact is in agreement with our findings. In this line, Idowu et al. [29] reported a mineral profile for salmon frames that is comparable to our data, with calcium and phosphorous as the major minerals, followed by sodium and potassium.

In general, all fish species are well-balanced regarding essential amino acids [27]. The EAA/NEAA ratio in cultured sea bream and sea bass muscles ranged from 0.9 to 1, depending on variations in the raw material composition [30]. The more abundant nonessential amino acids were glutamic acid, glycine, and aspartic acid in all residues of both species and in each residue. For heads and bones in sea bass and sea bream, the main essential amino acids were arginine, leucine, and lysine. These results are in accordance with [31], in which the essential and nonessential amino acid composition showed a similar composition. 

Amino acid profile of residues has importance in the peptide profile, which directly affects the antioxidant capacity [32,33], because it has been demonstrated that antioxidative peptides often ranged from 500 to 1800 Da, along with included hydrophobic amino residues, such as Pro, His, Tyr, Trp, Met, and Cys, in their sequences [34]. Certainly, bibliography reveals that peptides comprising hydrophobic residues, such as His, Met, and Cys, are determinant in the neutralization activity of hydroxyl radical [35]; meanwhile, the occurrence of aromatic residues (Tyr and Phe) provide the peptide the greater capacity to donate electrons [32,33]. During a PEF treatment, peptides and amino acids are produced by the protein proteolysis. It has been demonstrated that smaller peptides from fish products improve the capacity of electron donors and their reaction with free radicals stopping chain reactions and influencing higher angiotensin converting enzyme inhibitor activity [36].

### 4.2. Antioxidant Activity of Extracts from Residues

The high antioxidant capacity found in the extracts obtained by PEF reached values of 389.62 µg Trolox/g sample with DPPH assay (Figure 2), suggesting an intense release of antioxidant compounds from the matrix. The PEF technology is being currently tested to extract compounds from vegetable products [37,38,39]. Pulses act at the cellular level, breaking the cell membrane structure [40], allowing the release of the intracellular content. In this sense, Barba et al. [41] indicated that the application of PEF enhances the rate and the extraction efficiency of high-added-value compounds.

However, literature about the use of PEF treatment in fish residues is inexistent. Overall, PEF is one of the emerging techniques to preserve liquid foods (milk, yoghurt, juices, etc.), but it is rarely applied to solid foods. There are several authors who suggested that PEF could improve the release of peptides from foods [8,42]. Indeed, Ghosh et al. [8] extracted functional proteins with antioxidant properties from chicken wastes, employing PEF technology and using a cycle starting with short pulses of high voltage, followed by long pulses of low voltage. There are other studies which suggest that the PEF could generate a slight proteolysis in the muscles. Indeed, Bhat et al. [43] reported changes in the proteolysis patterns of troponin-T and desmin during the ageing of beef muscles previously treated with PEF. Therefore, it seems that the treatment with PEF could improve the extraction of compounds with antioxidant capacity from the extracts used in this study by two mechanisms. Firstly, the release of compounds from inside of cells such as endogenous antioxidants, and secondly, the proteolysis induced by PEF could provoke the peptide bonds’ breakage in the proteins, producing antioxidant peptides.

The different antioxidant activity found in the residues with methanol and water revealed the presence of compounds of different polarity in these by-products. Our results indicated that water enhanced the antioxidant activity, suggesting that compounds with higher polarity have more antioxidant capacity. Likewise, Agregán et al. [44] also observed an increase on the extraction of polyphenols from the algae *Bifurcaria bifurcata*, using polar solvents.

Concerning the possibility of use fish residues, it should be noted that our values for sea bream and sea bass residues were superior than values reported for sea bream and sea bass fillets (30 and 10 µg Trolox/g sample, respectively) reported by [45]. Other authors have separated bioactive peptides from fish residues in order to evaluate the antioxidant capacity of each peptide after a purification step [46]. This suggest that the differences of antioxidant capacity may be due to different peptides produced by PEF treatment. Other enzymatic treatments were widely assayed to increase the antioxidant compounds in the mixture, as reviewed Pérez-Gálvez et al. [47].

Regarding type of residue, gills have also demonstrated to be an important source of bioactive compounds with antioxidant capacity. Indeed, Lin et al. [48] obtained antioxidant peptides from the carp gills through enzymatic hydrolysis that they were used to decrease protein oxidation in surimi. Concerning heads and bones, previous studies have demonstrated their possibilities as a source of natural antioxidants. Certainly, protein hydrolysates from head of skipjack tuna (*Katsuwonus pelamis*) [49] or those from the backbones of sheela (*Sphyraena barracuda*) and ribbon fishes (*Lepturacanthus savala*) [50] displayed strong antioxidant activity. In this study, the higher antioxidant capacity shown by the gills extract with respect to heads and bones could be due to higher concentration of peptides whose activity increased as their molecular weight decreased [48]. It is known that the treatment with PEF can hydrolyze proteins, and the proteolysis of fish residues promotes the breaking of peptide bonds. Moreover, the different findings showed by the antioxidant capacity assays used in the present study could be due to the different chemical base of each assay [51]. From these data, the most conclusive results were obtained from DPPH, which is the most used scavenging antioxidant assay worldwide.

## 5. Conclusions

The present research was carried out to evaluate the use of PEF technology to improve antioxidant extracts from gills, heads, and bones of sea bream and sea bass. The findings of the study revealed that water extraction assisted by PEF improved the antioxidant capacity of extracts with respect to water or methanol extracts. The antioxidant capacity of sea bream residues was proven by DPPH and ABTS and FRAP tests that indicated superior values than those obtained from sea bass residues. However, ORAC values were higher in methanolic extracts. For DPPH assay, extracts using water assisted by PEF from heads, bones, and gills yielded significant increases of 35.8%, 68.6%, and 33.8% for sea bream and 60.7%, 71.8%, and 22.1% for sea bass, respectively, with respect to water extracts. The findings of the present study suggest that PEF would be an environmentally friendly and economic choice for antioxidant-extract production from low-value fish residue. In the further research studies, experimental designs with a stronger hydrolysis should be addressed, as well as a deeper level of purification and identification of the extracts.

## Figures and Tables

**Table 1:** Chemical composition and mineral profile of residues (gills, heads, and bones) from sea bass and sea bream.

Chemical Composition and Mineral Profile	Sea Bass			Sea Bream			SEM	Species	Residue	Species × Residue
	Gills	Heads	Bones	Gills	Heads	Bones				
Water (%)	62.37^a1^	58.86^b1^	51.6^c1^	55.23^B2^	52.51^C2^	57.63^A2^	0.29	<0.001	<0.001	<0.001
Fat (%)	14.00^b1^	13.94^b1^	20.20^a1^	21.55^A2^	22.10^A2^	17.12^B2^	0.21	<0.001	0.201	<0.001
SFA	26.27^a1^	21.76^b1^	21.75^b1^	20.15^B2^	20.71^A2^	20.84^A2^	0.037	<0.0001	<0.0001	<0.0001
MUFA	46.02^a1^	43.45^c1^	44.19^b^	47.61^A2^	43.45^B2^	44.19^B^	0.045	<0.0001	<0.0001	<0.0001
PUFA	26.98^c1^	33.72^a^	32.96^b1^	31.17^B2^	33.76^A^	33.75^A2^	0.067	<0.0001	<0.0001	<0.0001
LCn3	11.79^a1^	10.22^b1^	8.87^c1^	7.43^C2^	9.77^A2^	9.34^B2^	0.049	<0.0001	<0.0001	<0.0001
n3	14.68^a1^	14.20^b1^	12.92^c1^	11.90^C2^	14.03^A2^	13.58^B2^	0.053	<0.0001	<0.0001	<0.0001
n6/n3	0.83^c1^	1.37^b1^	1.55^a1^	1.62^A2^	1.40^C2^	1.48^B2^	0.005	<0.0001	<0.0001	<0.0001
Protein (%)	16.58^a1^	15.48^b1^	14.24^c1^	13.92^B2^	12.91^C2^	16.39^A2^	0.087	<0.001	<0.001	<0.001
Ash (%)	5.57^c1^	9.96^a^	7.52^b^	6.44^B2^	9.14^A^	6.23^B^	0.15	0.180	<0.001	0.005
Ca	1382.62^c1^	2507.15^a^	2093.26^b^	1873.24^B2^	2389.24^A^	1618.82^B^	41.69	0.685	<0.001	<0.001
Fe	1.22^a1^	0.28^c1^	0.51^b1^	2.15^A2^	0.44^C2^	0.69^B2^	0.02	<0.001	<0.001	<0.001
K	180.51^b1^	194.31^b^	262.73^a1^	134.94^C2^	184.52^B^	300.67^A2^	1.96	0.144	<0.001	<0.001
Mg	36.77^a1^	29.04^b^	24.98^c1^	47.90^A2^	28.04^B^	30.70^B2^	0.44	<0.001	<0.001	<0.001
Mn	500.58^a1^	266.82^b1^	270.37^b1^	585.07^A2^	211.13^B2^	206.76^B2^	11.39	0.612	<0.001	0.006
Na	250.51^a^	162.86^b^	96.03^c^	258.79^A^	159.30^B^	98.00^C^	2.04	0.587	<0.001	0.449
P	742.60^b1^	1277.00^a^	1166.36^a^	955.92^B2^	1312.27^A^	989.20^B^	20.68	0.567	<0.001	0.01
Zn	1.41^b1^	2.12^a1^	1.27^c^	2.12^A2^	1.71^B2^	1.39^C^	0.02	<0.001	<0.001	<0.001
Cu	0.09^a1^	0.03^b^	0.10^a^	0.17^A2^	0.04^B^	0.14^A^	0.009	0.017	<0.001	0.172

Minerals: Mn is expressed in µg/100 g, and the others minerals are expressed in mg/100 g. SEM is standard error of mean; mean values followed by a letter ^(a–c)^ for sea bass and ^(A–C)^ for sea bream display significant differences (*p* < 0.05), within each species; mean values followed by a number ^(1–2)^ display significant differences (*p* < 0.05) between residues; LCn3 = long chain n3.

**Table 2:** Amino acid profile of residues (gills, heads, and bones) from sea bass and sea bream.

Amino Acid	Sea Bass			Sea Bream			SEM	Species	Residue	Species × Residue
	Gills	Heads	Bones	Gills	Heads	Bones				
Asp	846.50	891.53^1^	875.14^1^	790.94^B^	695.72^B2^	1000.97^A2^	11.95	0.088	<0.0001	<0.0001
Ser	533.61^a1^	491.36^b1^	440.95^c1^	449.97^B2^	399.96^C2^	501.55^A2^	3.10	<0.0001	<0.0001	<0.0001
Glu	1321.941	1337.36^1^	1286.85^1^	1270.81^B2^	1073.06^C2^	1446.03^A2^	14.84	0.088	<0.0001	<0.0001
Gli	1447.95^a^	1171.91^b^	956.69^c^	1073.14	1118.67	1110.40	31.73	0.118	0.015	0.007
Ala	896.49^a1^	657.11^b1^	618.39^b1^	702.50^A2^	586.47 ^2^	688.02^A2^	7.50	<0.0001	<0.0001	<0.0001
Pro	949.47^a1^	677.66^b^	531.53^c1^	623.98^2^	625.12	626.28^2^	16.83	0.008	<0.0001	<0.0001
Tyr	254.75	293.60^1^	270.31^1^	228.99^B^	204.54^B2^	306.86^A2^	6.79	0.062	0.003	<0.0001
NEAA	6280.75^a1^	5520.56^b1^	4980.08^c1^	5140.34^B2^	4703.57^C2^	5680.13^A2^	24.12	<0.0001	<0.0001	<0.0001
His	374.00^a^	323.26^b1^	300.67^b1^	311.53^B^	276.50^C2^	379.58^A2^	3.82	0.194	<0.0001	<0.0001
Arg	185.28^c^	844.34^a1^	775.38^b1^	206.41^C^	701.37^B2^	870.50^A2^	6.33	0.486	<0.0001	<0.0001
Thr	694.30^a1^	453.15^b1^	445.73^b1^	567.39^A2^	386.41^C2^	516.28^B2^	5.80	0.001	<0.0001	<0.0001
Val	571.80^a^	471.88^b1^	454.74^b1^	494.67^A^	367.85^B2^	541.70^A2^	6.46	0.02	<0.0001	<0.0001
Met	n.d.	142.20	128.46^1^	n.d.	126.45	162.84^2^	6.20	0.620	<0.0001	<0.0001
Lys	672.73^b^	747.62^ab1^	797.34^a1^	722.25^B^	581.78 ^C2^	903.19^A2^	15.49	0.911	<0.0001	0.077
Iso	382.16	387.51^1^	378.43^1^	342.11^B^	277.68^B2^	450.37^A2^	7.80	0.105	<0.0001	<0.0001
Leu	663.83	637.02^1^	608.12^1^	611.99 ^B^	469.31^C2^	742.38^A2^	11.60	0.228	<0.0001	<0.0001
Phe	474.36^a^	431.84^ab1^	390.92^b1^	382.93 ^B^	315.45^C2^	462.85^A2^	6.38	0.01	<0.0001	<0.0001
EAA	4018.50^b^	4438.86^a^	4279.83^ab1^	3639.30 ^B^	3502.84^B2^	5029.73^A2^	48.25	0.058	<0.0001	<0.0001
EAA/NEAA	064^b^	0.80^a1^	0.86a	0.70^B^	0.74 ^B2^	0.88^A^	0.01	0.647	<0.0001	0.077

Amino acids: mg/100 g of residue. SEM is standard error of mean; mean values followed by a letter ^(a–c)^ for sea bass and ^(A–C)^ for sea bream display significant differences (*p* < 0.05), within each species; mean values followed by a number ^(1–2)^ display significant differences (*p* < 0.05) between residues; NEEA = nonessential amino acids; EEA = essential amino acids; n.d. = not detected.
